# Modulating aging-associated phenotypes in keratinocytes using antisense oligonucleotides targeting glycogenes

**DOI:** 10.7150/ntno.135510

**Published:** 2026-10-09

**Authors:** Bhanubhong Prommalee, Chalermchai Pilapong

**Affiliations:** Laboratory of BioMolecular Imaging, Molecular and Cellular Biology, Department of Radiologic Technology, Faculty of Associated Medical Sciences, Chiang Mai University, Chiang Mai, Thailand.

**Keywords:** aging-like keratinocytes, glycogene, antisense oligonucleotides, milk-derived nanovesicle

## Abstract

Age-related keratinocyte dysfunction has been associated with altered glycosylation and dysregulated expression of several glycogenes, including *St6gal1*, *St3gal2*, and *Man1a*. Modulation of these aging-associated glycogenes may therefore represent a potential approach for investigating cellular aging phenotypes. In this study, an *in vitro* aging-like keratinocyte model exhibiting increased expression of aging-associated glycogenes was established. Antisense oligonucleotides (ASOs) targeting the upregulated glycogenes were designed and evaluated using different oligonucleotide chemistries and delivery systems. Target gene expression, lectin-based glycan profiles, Lgr6 expression, and wound closure were assessed following ASO treatment. Among the ASOs tested, phosphorodiamidate morpholino (PMO)-linked *Man1a* ASOs demonstrated greater suppression of *Man1a* mRNA than ASOs targeting *St6gal1* or *St3gal2*. Delivery of PMO-*Man1a* ASOs using milk-derived nanovesicles (NV) was associated with the greatest reduction in *Man1a* mRNA levels compared with commercial transfection reagents. Although no significant changes were detected in ConA-reactive mannose-rich glycans, treatment was associated with an increased proportion of Lgr6^+^ cells and accelerated wound closure in aging-like keratinocytes. Overall, this study provides proof-of-concept evidence that NV-mediated delivery of glycogene-targeting ASOs can modulate selected aging-associated phenotypes in keratinocytes.

## Introduction

The skin serves as a primary protective barrier for internal tissues and organs against external insults, including mechanical and chemical agents, and plays a crucial role in maintaining homeostasis by regulating water balance and body temperature through the coordinated activity of cells and micronutrients. In everyday life, individuals are continually exposed to environmental hazards such as ultraviolet radiation, fumes, and chemical substances. Skin aging is widely recognized as an inevitable biological process characterized by progressive loss of elasticity, reduction in epidermal thickness, and deterioration of barrier function 1. Glycosylation has emerged as a key contributor to the aging process, given its involvement in multiple biological mechanisms, including alterations in glycoconjugate patterns, glycol-redox interactions, and age-related genetic dysregulation 2-4. Accumulating evidence links skin aging to glycosylation-dependent glycan remodeling, as reflected by age-associated changes in gene expression and aging phenotypes. Notably, within the interfollicular epidermis and hair follicles—regions enriched in stem cell and differentiation markers—epidermal stem cells display distinct glycan profiles in aged mice compared with their younger counterparts. Upregulated glycogenes, including *St6gal1*, *St3gal2*, and *Man1a*, which are associated with glycan alterations in aged epidermal stem cells, have been identified. Overexpression of these glycogenes impairs regenerative capacity and compromises stem cell function 5. These observations underscore a strong association between glycosylation and stemness, highlighting a critical biological crosstalk that contributes to skin aging and may represent a promising therapeutic target for mitigating age-related skin deterioration.

Given the need for highly specific target modulation, nucleic acid-based therapies have been extensively investigated 6. These therapies offer several advantages, including high efficiency, strong target specificity, reduced adverse effects, and a reduced likelihood of inducing drug resistance. Moreover, integrating nucleic acid-based therapy with other therapeutic strategies enables the simultaneous modulation of multiple molecular targets, thereby synergistically enhancing therapeutic efficacy 7. While direct DNA manipulation raises profound ethical concerns in human research, RNA-based interventions have emerged as a preferable alternative. RNA-targeting technologies, particularly oligonucleotide-based regulation of RNA targets through mechanisms such as pre-mRNA splice switching, inhibition of mRNA translation, and small interfering RNA-mediated silencing. Although these strategies demonstrate high specificity in mRNA degradation, their clinical application remains limited by inefficient intracellular uptake 6. Antisense oligonucleotides (ASOs) represent a prominent RNA-targeting modality capable of selectively eliminating undesirable mRNA transcripts. ASOs regulate nucleolar and/or cytoplasmic mRNA by binding to complementary RNA sequences via Watson-Crick base pairing. Two principal mechanisms of action have been identified: RNase H-mediated cleavage of target mRNA and steric hindrance that interferes with RNA processing or translation. The functional outcome of ASO activity depends on both the target sequence and the specific chemical composition of the ASO 8. Furthermore, chemical and structural modifications can be incorporated to enhance ASO stability and pharmacokinetic properties. Despite these advances, the application of ASO-based therapies in the context of skin aging remains largely unexplored.

Based on the considerations outlined above, this study focuses on the application of a precise RNA-regulatory strategy using antisense oligonucleotides (ASOs) in aging keratinocytes. In addition, the study evaluates the capacity of ASOs to modulate aging-associated phenotypes. We further aim to optimize the efficacy of ASOs targeting upregulated glycogenes through nanovesicle delivery, with the goal of establishing a promising delivery platform for ASO-based precision medicine in the context of skin aging.

## Methods

### Design, prediction, and synthesis of antisense oligonucleotides

Antisense oligonucleotides (ASOs) were designed using bioinformatic tools. The University of California, Santa Cruz Genome Browser (UCSC Genome Browser) was used to obtain mRNA sequences corresponding to exon target regions of each glycogene. For each target, 200 base pairs upstream and downstream of the coding sequence exons were selected and submitted for oligonucleotide design using the eSkip-Finder web tool. Target accessibility and secondary RNA structure were predicted using the RNAfold web server (ViennaRNA Web Services). In addition, the feasibility of ASO-mRNA interactions were assessed using the AlphaFold3 server (DeepMind).

ASOs including phosphodiester (PO), phosphorothioate (PS), and phosphorodiamidate morpholino oligomer (PMO) linkages were synthesized commercially (Integrated DNA Technologies and Gene Tools). Fluorescently labeled ASOs were also generated to evaluate intracellular accumulation and cellular targeting efficiency. The synthesized ASOs included Control-PO, Control-PS, Control-PMO, *St6gal1*-PO, *St3gal2-*PO, *Man1a-*PO, *St6gal1*-PS, *St3gal2*-PS, *Man1a*-PS, *St6gal1*-PMO, *St3gal2*-PMO, *Man1a*-PMO, FITC-*St6gal1*, and Cy3-*Man1a*.

### Cell lines and cell culture

Human epidermal keratinocytes (HaCaT) were maintained in Dulbecco's Modified Eagle's Medium (DMEM; Caisson Laboratories) supplemented with 10% fetal bovine serum (FBS) and 1% penicillin-streptomycin solution at 37 °C in a humidified atmosphere containing 5% CO_2_. For the healthy cell model, cells in the logarithmic growth phase were used, whereas post-confluent HaCaT culture was selected as an experimentally established model of stress-induced aging-like keratinocytes 9.

### Cellular accumulation of antisense oligonucleotides

Healthy keratinocytes were seeded at a density of 1.0 × 10^5^ cells/mL in 12-well culture plates and incubated overnight. The cells were subsequently treated with 1.0 nmol of FITC-labeled *St6gal1* ASO or Cy3-labeled *Man1a* ASO for 24 h. For selected experiments, the Cy3-labeled ASOs were pre-incubated with milk-derived NV for 5 min at loading ratios of ASO:NV prior to treatment. Intracellular uptake was assessed after 24 h treatment by fluorescence microscopy and flow cytometry. Following treatment, cells were washed and analyzed for intracellular ASO accumulation using fluorescence microscopy (Nikon Eclipse Ts2) and flow cytometry (Beckman Coulter CytoFLEX).

### Quantitative reverse transcription polymerase chain reaction (qRT-PCR)

To evaluate the efficiency of ASOs in modulating target mRNA expression, quantitative reverse transcription polymerase chain reaction (qRT-PCR) was performed. Aging-like keratinocytes were seeded at a density of 1.0 × 10^5^ cells/mL in 12-well culture plates and incubated overnight. Cells were then treated with various ASOs, with or without transfection agents, for 24 h. Milk-derived NV and a commercially available transfection reagent (jetPRIME) were used as delivery systems in this study.

Total RNA was isolated using the RNeasy Micro Kit (QIAGEN; #74104). Complementary DNA (cDNA) was synthesized from 5 ng of total RNA using a thermocycler (SensoQuest). Real-time PCR was conducted using iTaq Universal SYBR Green Supermix (Bio-Rad) on a LightCycler 96 system (Roche), according to the manufacturers' instructions.

The primer sequences used in this study were as follows: *St6gal1* forward, AGCACCCAGGACCCCCACAG; *St6gal1* reverse, ACATGGTCCCGGAGGTGGCA; *St3gal2* forward, CTGGATGCTGGGACCTAC; *St3gal2* reverse, GCTACTTGGAAGGCTGAGG;* Man1a* forward, GCAGTGGAACTTGGGGTAAA; *Man1a* reverse, TTCAGCAAAGATGGGGTTTC; *GAPDH* forward, TGGCATATCTCTTATTAAGGGGG; *GAPDH* reverse, ACTTCTCATCAGCCACCTCG. Relative expression levels of *St6gal1* and *Man1a* following ASO treatment were quantified using *GAPDH* as the internal reference gene.

### Lectin-binding assay

Lectin-binding assay was primarily visualized to assess aging-associated alterations in glycosylation. Alexa Fluor 594-conjugated wheat germ agglutinin (WGA; iFluor, AAT Bioquest; #25550) and Alexa Fluor 488-conjugated concanavalin A (ConA; iFluor, AAT Bioquest; #25580) were used to detect sialic acid and α-D-mannosyl residues on the cell membrane, respectively. Initially, healthy and aging-like keratinocytes were stained with WGA and ConA to evaluate differences in lectin-recognized glycan signatures associated with the aging-like phenotype.

To evaluate the efficacy of ASO treatment, aging-like keratinocytes were seeded at a density of 1.0 × 10^5^ cells/mL in 12-well culture plates and incubated overnight. Cells were then treated for 24 h with pure milk-derived NV, pure jetPRIME reagent (Pure Jet), PMO-*Man1a* ASOs, PMO-*Man1a* ASOs delivered using milk-derived NV, or jetPRIME. The final concentration of ASOs was 0.1 nmol. The loading ratio of ASO to the transfection reagent was 0.1 nmol:5 μL for both milk-derived NV and jetPRIME. Following treatment, cells were washed with phosphate-buffered saline (PBS) and fixed with 4% paraformaldehyde at 37 °C for 15 min. Fixed cells were incubated in PBS containing 1% bovine serum albumin (BSA) and the indicated lectins for 1 h at 4 °C in the dark, followed by two washes with PBS. Lectin binding expression was subsequently analyzed using fluorescence microscopy and flow cytometry.

### Lgr6^+^ expression analysis

Aging-like keratinocytes were seeded at a density of 1.0 × 10^5^ cells/mL in 12-well culture plates and incubated overnight. Cells were then treated for 24 h with pure milk-derived NV, pure jetPRIME reagent (Pure Jet), PMO-*Man1a* ASOs, PMO-*Man1a* ASOs delivered using milk-derived NV, or PMO-*Man1a* ASOs delivered using jetPRIME. The final concentration of ASOs was 0.1 nmol. The loading ratio of ASO to the transfection reagent was 0.1 nmol:5 μL for both milk-derived NV and jetPRIME. Following treatment, cells were trypsinized and fixed with 4% paraformaldehyde in phosphate-buffered saline (PBS) for 15 min at 4 °C. After washing with PBS, cells were incubated with an anti-Lgr6 primary antibody (Sigma) diluted 1:200 in PBS containing 1% bovine serum albumin (BSA) for 1 h at room temperature in the dark. Cells were then washed twice with PBS and incubated with a goat anti-rabbit IgG Alexa Fluor 488-conjugated secondary antibody (Thermo Fisher Scientific) diluted 1:500 in 1% BSA for 1 h at room temperature in the dark, followed by two additional washes with PBS. Lgr6 expression was analyzed using flow cytometry.

### Scratch assay

Aging-like keratinocytes were seeded at a density of 1.0 × 10^5^ cells/mL in 12-well culture plates and incubated overnight to reach confluence. A linear scratch was then created at the center of each well using a P200 pipette tip. After washing with phosphate-buffered saline (PBS) to remove detached cells and debris, the cultures were treated with scramble PMO ASO, PMO-*Man1a* ASOs, or PMO-*Man1a* ASOs delivered using NV for the indicated time periods. The final concentration of ASOs was 0.1 nmol. The loading ratio of ASO to the transfection reagent was 0.1 nmol:5 μL for milk-derived NV. Wound closure was monitored using an inverted microscope at 0, 24, and 48 h. Quantitative analysis of wound closure was performed using ImageJ software.

### Statistical analysis

Statistical analyses were performed using GraphPad Prism version 8.0 (GraphPad Software, San Diego, CA, USA). Data are presented as mean ± standard deviation (SD), unless otherwise indicated. Details of the statistical tests and the number of biological replicates used for each dataset are provided in the corresponding figure legends.

## Results

Antisense oligonucleotides (ASOs) were designed using a site-specific strategy based on sequence alignment to identify regions with high predicted complementarity between ASOs and target mRNA. Multiple open-source bioinformatic tools were employed during the design process. Specifically, the UCSC Genome Browser and the eSkip-Finder web platform were used to identify suitable target regions. ASO binding sites were determined at both the exon and chromosomal levels. In addition, the Gibbs free energy change (ΔG), a thermodynamic parameter that reflects the spontaneity of molecular interactions under constant temperature and pressure, was considered a critical criterion for ASO localization. Among candidate sites with negative ΔG values, those with the lowest (most negative) ΔG were selected, indicating the highest predicted binding stability. Subsequently, the eSkip-Finder platform generated 25-nucleotide ASO sequences complementary to each targeted exon (Table 1).

ASO structures were further predicted using bioinformatic tools, including the ViennaRNA Web Service and AlphaFold. Structural predictions revealed distinct conformational features for each ASO strand based on sequence alignment. Specifically, key-like, cylindrical, and circular configurations were observed for *St6gal1*, *St3gal2*, and *Man1a* ASOs, respectively (Figure 1A). In addition, the ViennaRNA Web Service was used to identify potential ASO-mRNA interaction regions. Predicted RNA secondary structures exhibited stem-loop (hairpin) and multibranch loop motifs, with ASO binding sites predominantly located within double-stranded regions adjacent to multibranch loops (Figure 1B). Predictions of ASO-mRNA interactions were further refined using the AlphaFold3 web server to generate three-dimensional models illustrating ASO-mRNA binding. These schematic representations facilitated visualization of the most probable binding conformations and interaction interfaces (Figure 1C). Notably, *Man1a* ASOs exhibited higher per-atom confidence scores at specific interaction sites compared with the other ASOs, indicating more stable predicted binding.

Consistent with these *in silico* predictions, fluorescence microscopy and flow cytometry analyses demonstrated significantly higher signal intensities of FITC-labeled *St6gal1* and Cy3-labeled *Man1a* ASOs in HaCaT cells following 24 h incubation compared with untreated controls, indicating efficient cellular uptake (Figure 2A).

The primary objective of this study was to develop a targeted strategy for modulating age-associated glycosylation biology in aging-like keratinocytes, with a particular focus on age-related glycogenes. Three glycogenes identified as significantly upregulated during aging were selected as key targets for investigation. Accordingly, this study employed an antisense oligonucleotide (ASO)-based approach to perturb aging-associated glycogenes in aging-like keratinocytes.

To understand the relevance of the aging model, appropriate conditions for inducing keratinocyte aging were carefully considered. Multiple factors have been reported to contribute to cellular aging, including oxidative stress, chronological aging, inflammation, cellular damage, and extrinsic influences such as photoaging 11, 12. In the present study, chronological aging was adopted as an *in vitro* model, achieved by extending cell culture beyond the logarithmic growth phase. The validity of this aging model was assessed by comparing mRNA expression profiles between aging and healthy keratinocytes to determine whether age-related glycogene upregulation was recapitulated. As shown in Figure 2B, aging-like keratinocytes exhibited increased expression of *St6gal1* and *Man1a* compared with healthy cells. Consistent with these glycogene changes, lectin-binding assay demonstrated altered downstream glycosylation patterns. Flow cytometric analysis with wheat germ agglutinin (WGA) and concanavalin A (ConA) revealed increased sialic acid-containing glycans and decreased mannose-containing glycans in aging-like keratinocytes relative to healthy controls (Figure 2C). This observation is consistent with the critical role of age-associated glycosylation in epidermal stem cells during skin aging 5. Collectively, these findings indicate that the established aging model was inferred to age-associated upregulation of glycogenes, thereby providing a suitable platform for subsequent investigations into glycogene regulation in aging-like keratinocytes.

Because ASO properties can be modulated through diverse backbone chemistries, this study evaluated multiple ASO linkage types targeting aging-like keratinocytes with respect to efficacy and cytotoxicity. Phosphodiester (PO), phosphorothioate (PS), and phosphorodiamidate morpholino oligomer (PMO) linkages were compared, and the chemical structures of these ASOs are illustrated in Figure 3A. Cell viability was assessed to evaluate the potential cytotoxic effects of ASO treatment (1.0 nmol). A slight decrease in cell viability was observed across all treatment groups; however, these differences were not statistically significant. More than 50% of the cells remained viable under all experimental conditions, suggesting that the treatments were generally well tolerated (Figure S1).

To assess the efficacy of ASOs in suppressing glycogene expression, ASOs incorporating different linkage chemistries (Figure 3A) were evaluated by quantifying target mRNA levels. Consistent with earlier findings, *St6gal1* and *Man1a* mRNA expression levels were elevated in aging-like keratinocytes and were therefore selected for investigation. For *St6gal1*, none of the tested ASO linkages resulted in a reduction in mRNA expression (Figure 3B). Similarly, *Man1a* mRNA levels were largely unaffected by PO and PS ASOs; however, a notable reduction was observed following treatment with the PMO-*Man1a* ASO (Figure 3C).

Based on the preceding results, PMO-*Man1a* ASOs exhibited superior efficacy in suppressing target mRNA expression and were therefore selected for subsequent experiments. Genetic manipulation is commonly achieved using lipid nanoparticle-based delivery systems. In our previous work, we established a solvent-free method for generating milk-derived NV, which exhibited a small particle size, narrow size distribution, and a negative surface charge. The milk-derived NV demonstrated good biocompatibility, with no significant effects on cell viability, ROS generation in HaCaT cells, or immune activation in PBMCs. Moreover, milk-derived NV efficiently delivered diverse nucleic acid cargos, including single-stranded DNA, microRNA, and mRNA, into HaCaT cells. Collectively, these findings support milk-derived NV as a biocompatible, low-immunogenic, and readily prepared natural nanocarrier with favorable structural properties for nucleic acid delivery 13. To determine whether milk-derived NV was capable of delivering ASOs into cells, Cy3 labeled ASOs were directly incubated with FITC-labeled NV and used for subsequent cellular uptake experiments. Fluorescence imaging revealed stronger intracellular FITC signals (representing NV) and Cy3 signals (representing the ASO strand) in cells treated with NV-loaded ASOs than in those treated with free ASOs. Flow cytometric analysis confirmed significantly increased uptake of FITC-labeled ASOs in both the 5 μL and 10 μL NV groups. In contrast, only the 5 μL NV formulation significantly enhanced the uptake of Cy3-labeled ASOs compared with the No-NV control (Figure S2). Accordingly, the delivery efficiency of PMO-*Man1a* ASOs using NV was evaluated and compared with that of a commercially available transfection reagent (jetPRIME). As shown in Figure 4A, *Man1a* mRNA expression was significantly reduced in cells treated with PMO-*Man1a* ASOs delivered via NV compared with all other conditions, including untreated controls and the jetPRIME-mediated delivery group. These results indicate that NV-mediated delivery of PMO-*Man1a* ASOs achieved more efficient mRNA suppression than the commercial transfection reagent.

The potential of the designed ASOs to demonstrate lectin-binding expression was further examined. However, flow cytometric analysis revealed no significant changes in FITC-concanavalin A binding, corresponding to mannose-type glycans, across any treatment group compared with untreated controls (Figure 4B).

Given the established association between glycosylation and stemness, the capacity of PMO-*Man1a* ASOs to enhance cellular stemness was further evaluated. In this context, Lgr6^+^ expression analysis was primarily assessed by quantifying the expression of Lgr6⁺ cells. The results demonstrated that NV-mediated delivery of PMO-*Man1a* ASOs significantly increased both the proportion of Lgr6⁺ cells and overall Lgr6^+^ expression levels compared with control groups (Figure 4C-D). Furthermore, NV-mediated ASO delivery exhibited superior transfection efficiency relative to the commercial transfection reagent jetPRIME.

In general, wound healing is a critical process in tissue regeneration and is known to decline with aging. Accordingly, we investigated whether ASO treatment, particularly PMO-*Man1a* ASOs delivered via milk-derived NV, could enhance the migratory capacity of aging-like keratinocytes. A scratch wound assay was performed as a preliminary assessment of reparative cellular behavior *in vitro*. Wound closure was quantified at multiple time points from 0 to 48 h following treatment. The results demonstrated that PMO-*Man1a* ASOs delivered by milk-derived NV produced the greatest enhancement of reparative cellular behavior, as evidenced by the most pronounced reduction in scratch area over time compared with PMO-*Man1a* ASOs without milk-derived NV, control ASOs, and untreated controls. (Figure 4E).

## Discussion

The application of precision medicine for the correction of genetic defects remains challenging; however, accumulating evidence underscores the therapeutic potential of RNA-based interventions for precise modulation of gene expression. RNA-targeting strategies, including small interfering RNA (siRNA), antisense oligonucleotides (ASOs), and microRNA (miRNA), have demonstrated clinical promise in the treatment of genetic disorders and cancers 14. Among these approaches, ASOs are particularly advantageous due to their high sequence specificity, which is achieved through Watson-Crick base pairing with target mRNA, enabling selective and efficient transcript modulation.

Previous studies have highlighted the versatility of ASOs in disease contexts, particularly in oncology, where ASO-mediated inhibition of aberrant splicing has been shown to reduce alternative splicing events while limiting off-target toxicity in non-malignant cells 15. Furthermore, successful correction of pathogenic mutations in the COL17A1 gene via ASO-induced exon skipping demonstrates the feasibility of manipulating pre-mRNA processing to restore gene function 16. Together, these findings provide a strong rationale for extending ASO-based strategies beyond classical genetic diseases and cancers to age-related conditions, including skin aging, where precise regulation of gene expression may offer therapeutic benefit.

To investigate ASO-mediated genetic modulation in aged keratinocytes, the HaCaT cell line was employed as a well-established human keratinocyte model due to its genetic stability, reproducibility, and lower variability compared with primary keratinocytes. Prolonged post-confluent culture of HaCaT cells for five days resulted in significant upregulation of *St6gal1* and *Man1a* mRNA expression, consistent with previously reported aging-associated glycosylation changes 5. In contrast, *St3gal2* expression did not exhibit a similar upregulation pattern, suggesting gene-specific regulatory differences.

The discrepancy observed for *St3gal2* may reflect intrinsic differences between *in vitro* human keratinocyte models and *in vivo* mouse systems, including species-specific transcriptional regulation and context-dependent glycosylation pathways. These findings highlight the importance of model selection when interpreting age-related gene expression patterns and underscore the complexity of glycogene regulation in aging skin.

In this context, we focused on modulating skin aging-associated glycogenes, specifically sialyltransferase and mannosidase genes that have been shown to influence aged epidermal stem cell function 5. Targeting these genes using antisense oligonucleotides (ASOs) was selected as the primary genetic modulation strategy, as ASOs are well established for selectively downregulating target transcript levels. Accordingly, disruption of β-galactoside α2,6-sialyltransferase (*St6gal1*), which catalyzes terminal α2,6-sialylation of cellular proteins and is upregulated in aged interfollicular epidermal stem cells, was expected to result in reduced gene expression following ASO treatment. A similar regulatory outcome was anticipated for *Man1a*, a key mannosidase involved in glycan processing.

In the present study, delivery of antisense oligonucleotides (ASOs) targeting *Man1a* resulted in a significant reduction in *Man1a*-mRNA expression, with the greatest effect observed when the ASOs were delivered using NV. These findings indicate that the NV platform effectively facilitated intracellular delivery of the ASOs under the experimental conditions used. Although *Man1a*-targeting ASOs exhibited greater knockdown efficiency than *St6gal1*-targeting ASOs, the underlying basis for this difference remains uncertain. Potential contributing factors may include differences in target-site accessibility, ASO-mRNA interaction dynamics, intracellular trafficking, or sequence-specific properties of the oligonucleotides. However, these possibilities were not directly investigated in the present study and therefore remain speculative. In general, the mechanisms underlying ASO-mediated transcript suppression have been well characterized and include RNase H-dependent mRNA degradation, steric blocking near the 5′ untranslated region (UTR) or 3′ UTR, and modulation of pre-mRNA splicing through exon skipping 17. However, the observed reduction in *Man1a* transcript abundance does not necessarily indicate the canonical mechanism of PMO activity. Possible explanations include altered transcript processing, reduced transcript stability secondary to splice interference, or indirect regulatory effects.

With respect to ASO subclasses, phosphorothioate (PS) and phosphorodiamidate morpholino oligomer (PMO) linkages were evaluated to determine whether distinct backbone chemistries influenced target disruption efficiency. Among these, PMO-modified ASOs demonstrated superior efficacy in reducing *Man1a* mRNA levels compared with other linkage types. This enhanced performance may be attributed to the intrinsic properties of PMOs, including their strong and stable binding to complementary RNA via Watson-Crick base pairing, as well as their resistance to nuclease and protease degradation 17.

Lipid-based nanovesicles have been widely demonstrated to facilitate efficient gene delivery and exert promising biological effects across multiple studies 18, 19, particularly in the context of antisense oligonucleotide (ASO) delivery 20. Rather than developing a fully optimized nanocarrier platform, this proof-of-concept study sought to evaluate whether naturally milk-derived nanovesicle (NV) could serve as biologically compatible and economically feasible vehicles for ASO delivery. Recently, our NV illustrated satisfactory outcome of nucleic acid delivery, including single-stranded DNA, microRNA, and mRNA, into HaCaT cells 13. In the present study, we comparatively evaluated the transfection efficiency of milk-derived NV against a commercially available polymer-based transfection reagent, jetPRIME.

Our results showed that PMO-*Man1a* ASOs delivered via milk-derived NV were associated with a significantly greater reduction in *Man1a* mRNA levels than all other treatment conditions, including jetPRIME-mediated delivery. These findings suggest that the milk-derived NV formulation improved the functional performance of PMO-ASOs under the present experimental conditions. The observed modulation of *Man1a* transcript levels is consistent with successful intracellular delivery of the ASOs 21.

Despite the significant reduction in *Man1a* mRNA expression following PMO-*Man1a* ASO delivery via milk-derived NV, no significant changes were detected in mannose-rich glycan expression using ConA lectin staining. These findings indicate that the transcript-level modulation observed in the present study was not accompanied by detectable alterations in this lectin-based glycosylation readout. One possible explanation is the inherent complexity of N-glycan biosynthesis, which is regulated by coordinated interactions among multiple glycosyltransferases, glycosidases, glycan transporters, and substrate availability rather than by the activity of a single enzyme alone 22. In addition, ConA recognizes only a subset of mannose-containing glycans and therefore may not be sufficiently sensitive to detect subtle or site-specific changes in glycan processing. Consequently, the present findings do not establish glycosylation remodeling as the mechanism underlying the observed cellular responses. More comprehensive analyses, including *Man1a* protein expression, enzymatic activity, and high-resolution glycomic profiling, will be required to determine whether *Man1a* modulation results in biologically meaningful alterations in glycosylation.

Notably, NV-mediated delivery of PMO-*Man1a* ASOs was associated not only with effective modulation of *Man1a* mRNA expression but also with increased expression of the epidermal progenitor-associated marker Lgr6^+^. Specifically, a significantly higher proportion of Lgr6^+^ cells was observed compared with the control groups. Although Lgr6^+^ has been reported to be associated with epidermal progenitor cell populations, its expression alone is insufficient to establish enhanced stemness or stem cell function. In sum up, these observations suggest that targeted modulation of aging-associated glycogenes may influence cellular phenotypes linked to epidermal regeneration; however, the underlying molecular mechanisms and their relationship to stemness remain to be established through future functional and mechanistic studies, especially, confirmation using additional stemness markers; e.g., K15, ITGA6, SOX9, p63.

In addition to molecular markers, wound closure capacity is associated with a skin rejuvenation-related phenotype. Nanovesicle-delivered PMO-Man1a ASOs significantly enhanced the wound closure capacity of aging-associated keratinocytes. Together, these findings suggest that ASO-mediated modulation of Man1a expression in aging-associated keratinocytes may have potential therapeutic relevance for promoting a skin rejuvenation-associated phenotype.

## Conclusion

This study provides proof-of-concept evidence that antisense oligonucleotides (ASOs) targeting aging-associated glycogenes can modulate selected aging-associated phenotypes in an *in vitro* aging-like keratinocyte model. Delivery of PMO-*Man1a* ASOs using milk-derived NV was associated with significant modulation of *Man1a* mRNA expression compared with conventional transfection. In parallel, treated cells exhibited accelerated wound closure and an increased proportion of Lgr6^+^ cells, findings that are consistent with a regenerative-associated phenotype. Despite effective modulation of *Man1a* transcript levels, no significant changes were detected in ConA-reactive mannose-rich glycans, indicating that the present study does not establish glycosylation remodeling as the mechanism underlying the observed phenotypic responses. Instead, the findings suggest that modulation of aging-associated glycogenes may influence keratinocyte behavior through mechanisms that remain to be elucidated.

Overall, this study supports the feasibility of milk-derived NV as a delivery platform for glycogene-targeting ASOs and provides an experimental foundation for further investigation of glyco-targeted nucleic acid therapeutics in skin aging. Future studies incorporating comprehensive senescence characterization, *Man1a* protein and enzymatic measurements, high-resolution glycomic profiling, complementary stem-cell markers, and *in vivo* validation will be essential to define the underlying mechanisms and evaluate the translational potential of this approach.

## Supplementary Material

Supplementary figures.

## Figures and Tables

**Figure 1:**
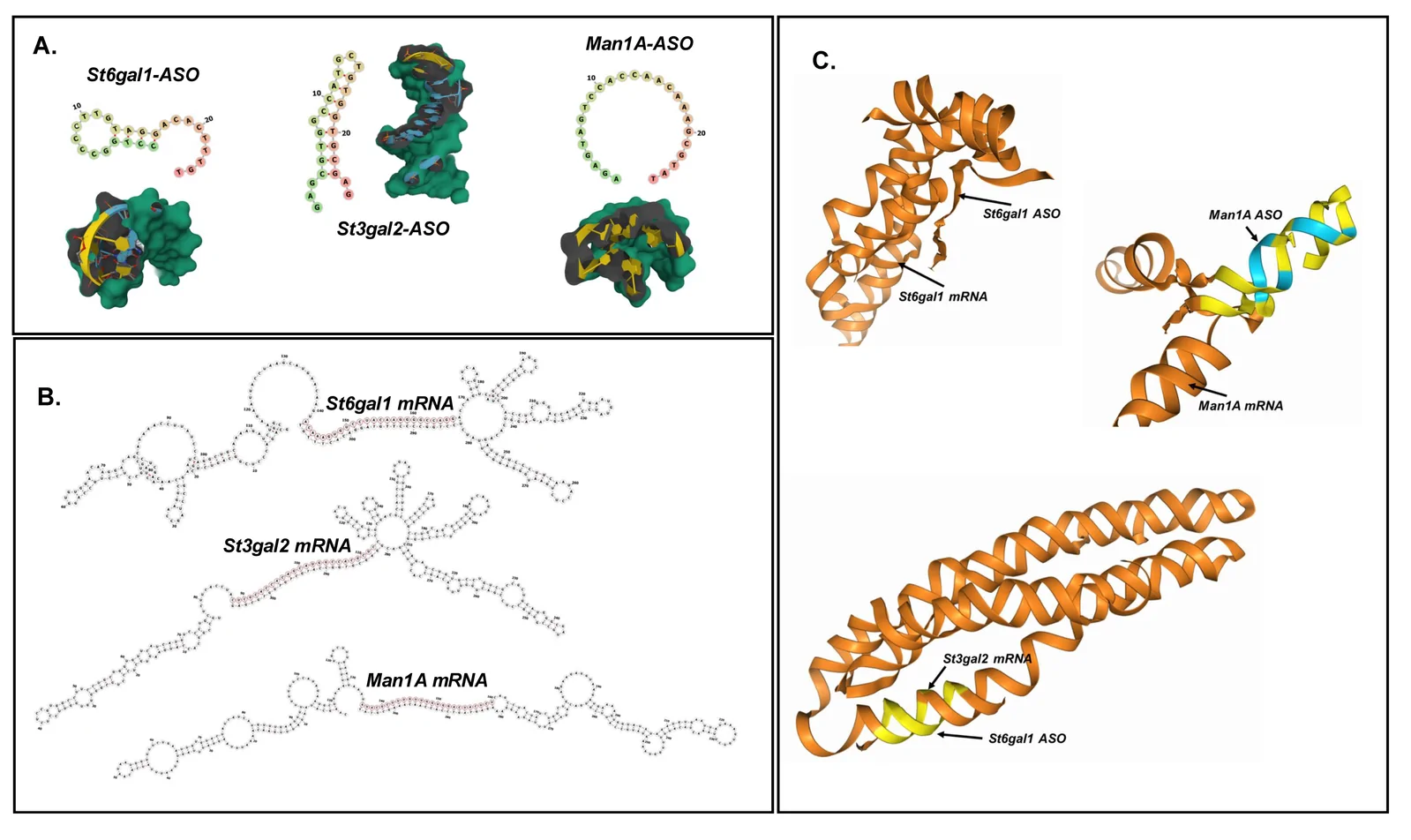
(A) 2D structure and 3D structure of different ASOs predicted by Vianna RNA Web and AlphaFold3, respectively. (B) Predicted active sites (label in red) of ASO attachment in the mRNA secondary structures, identified by ViennaRNA webservice. (C) The complementary interaction between ASO and its mRNA sites predicted by per-atom confident levels using AlphaFold3 Server. Blue represented the areas providing 70-90%, yellow accounted for 50-69%, and orange accounted for less than 50% of confident levels of prediction upon ASOs-mRNA specific interactions.

**Figure 2:**
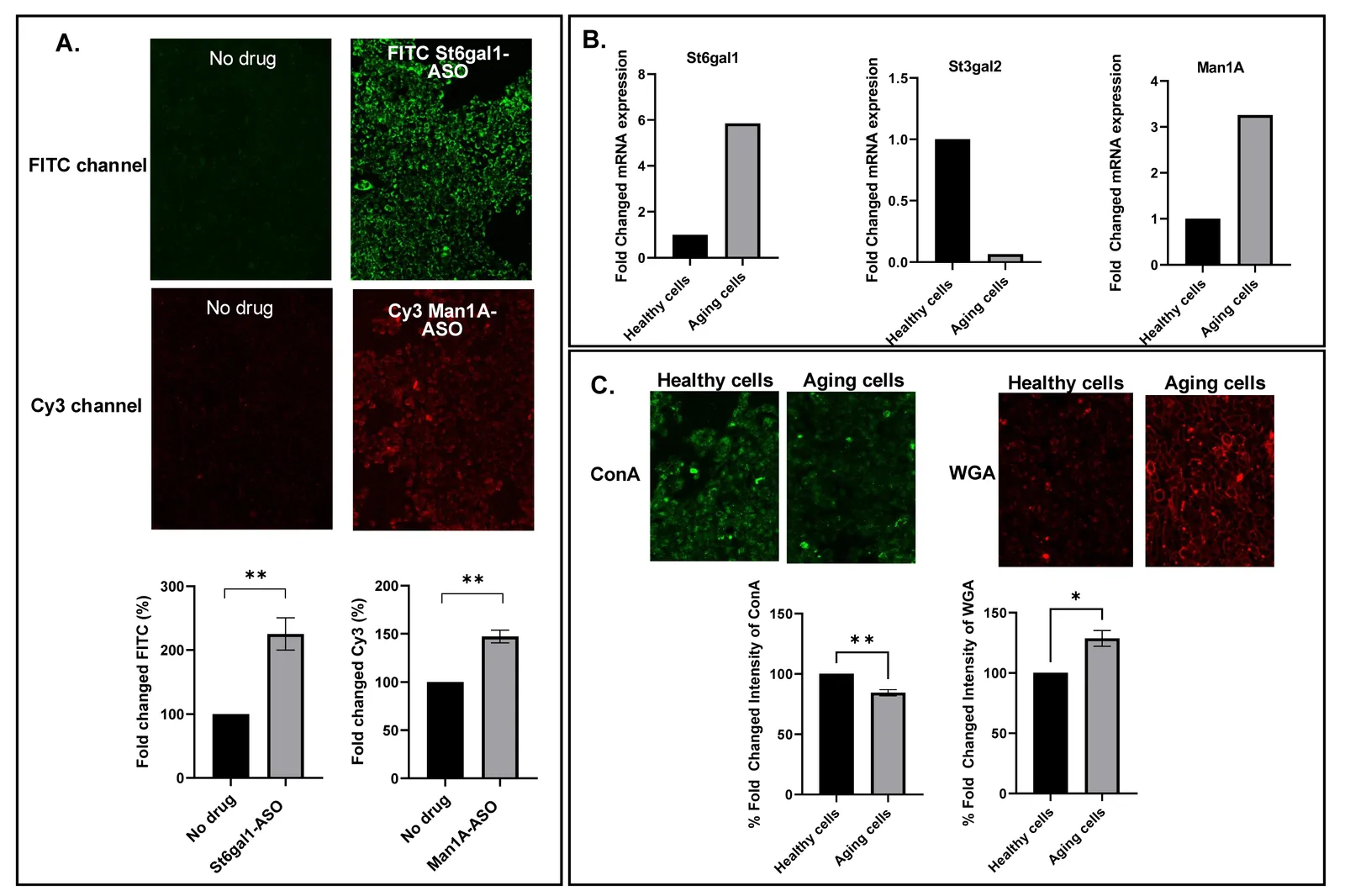
(A) Fluorescence imaging and flow cytometry analysis of HaCaT cells being incubated with FITC labeled *St6gal1* and Cy3 labeled *Man1A*-ASOs (1.0 nmol) for 24 hours (n=3, **p < 0.01 by un-paired t test). (B) Glycogenes expressions induced by culture-induced aging model, determined by qRT-PCR (n=1). (C) Glycan fluorescence imaging and semiquantitative analysis of glycan expression determined by flow cytometry of healthy and aging-like cells using glycan probes (ConA and WGA) (n=3, *p < 0.05, **p < 0.01 by un-paired t test).

**Figure 3:**
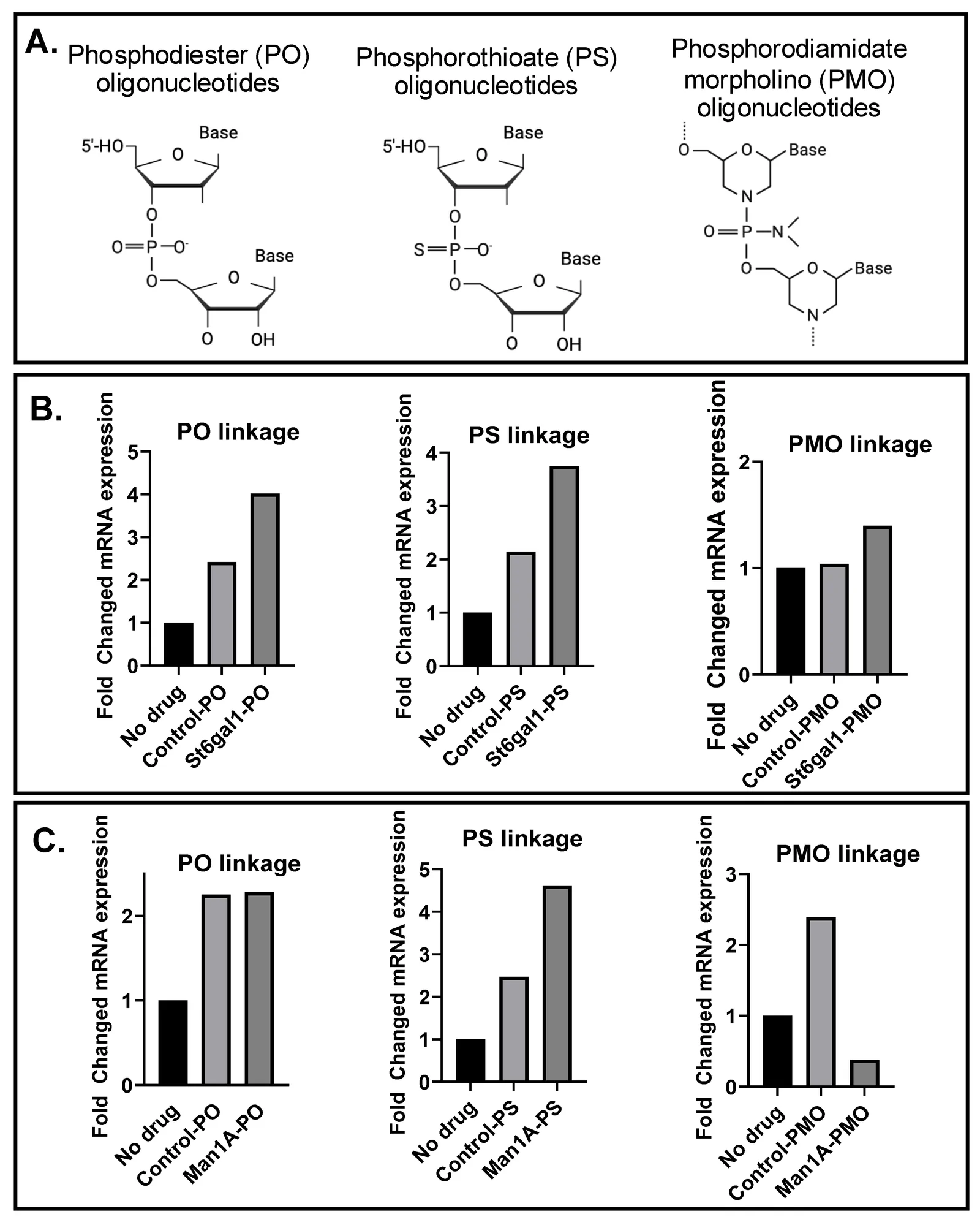
(A) Linkage chemistries of ASOs used in this study. (B) mRNA levels following treatment with *St6gal1*-targeting ASOs (1.0 nmol) containing phosphodiester (PO), phosphorothioate (PS), or phosphorodiamidate morpholino oligomer (PMO) linkages (n=1). (C) mRNA levels following treatment with *Man1a*-targeting ASOs (1.0 nmol) containing phosphodiester (PO), phosphorothioate (PS), or phosphorodiamidate morpholino oligomer (PMO) linkages (n=1). Control: cells treated with a sequence-scrambled ASO.

**Figure 4:**
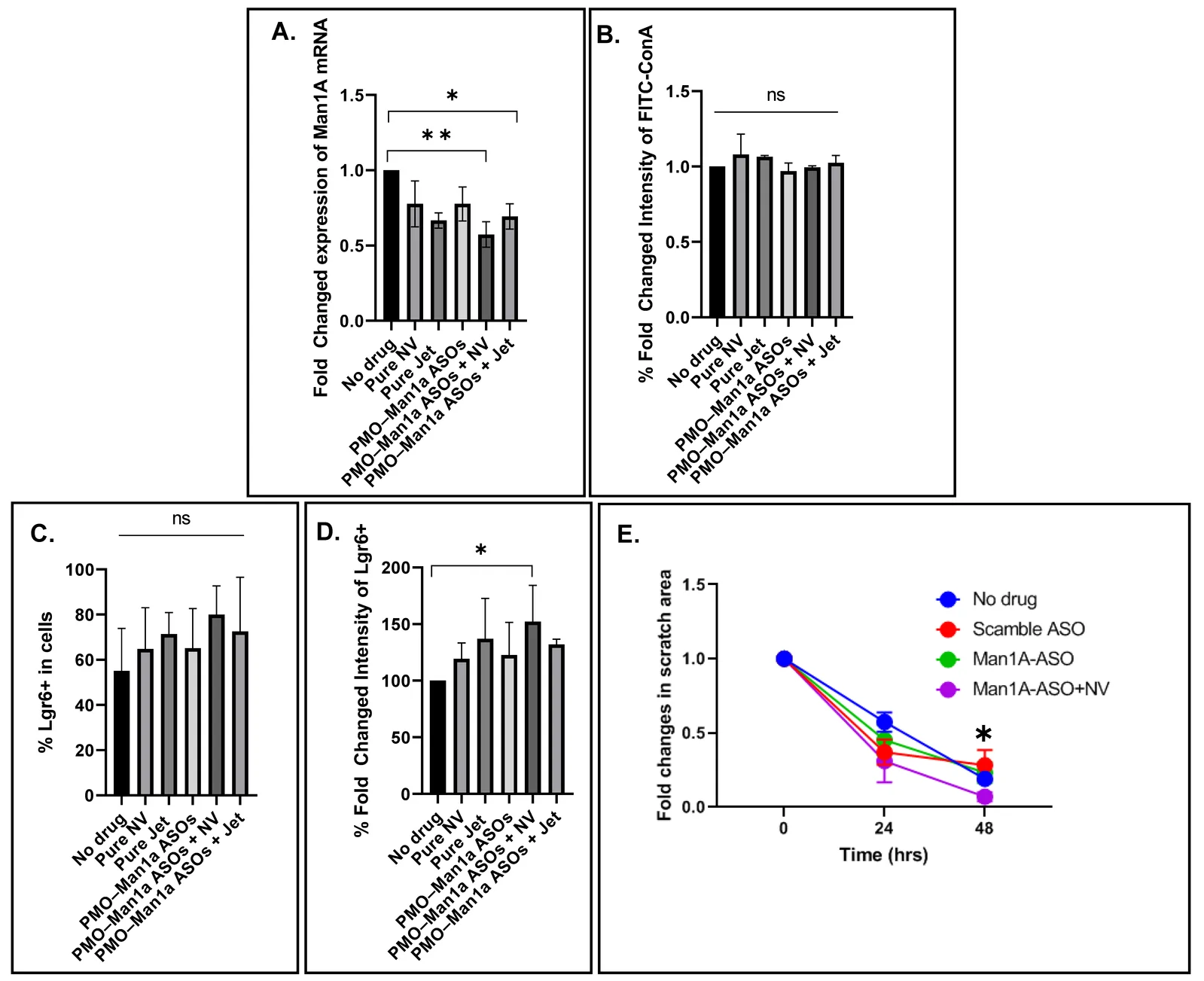
(A) Relative Man1a mRNA expression in aging-like keratinocytes following treatment with Man1a ASO (0.1 nmol) with or without milk-derived nanovesicle (NV)-mediated delivery and the indicated control conditions. (B) Expression of mannose-type glycans, determined by FITC-concanavalin A (ConA) fluorescence, following treatment with Man1a ASO (0.1 nmol) with or without NV-mediated delivery and the indicated control conditions. (C) Proportion of Lgr6⁺ cells and (D) Lgr6^+^ fluorescence intensity in aging-like keratinocytes following treatment with Man1a ASO (0.1 nmol) with or without NV-mediated delivery and the indicated control conditions. (E) Wound closure of aging-like keratinocytes following treatment with Man1a ASO with or without NV-mediated delivery and the indicated control conditions. Experiments were performed independently in triplicate (n = 3). ns, not significant; *p < 0.05; **p < 0.01 (one-way ANOVA followed by Fisher's LSD post hoc test). NV: milk-derived nanovesicle; Jet: JetPRIME.

**Table 1:** Antisense oligonucleotides used in the study.

Gene	Location	ASO Sequences	dG
St6gal1	chro3, ex 4	CCTGGCCCCTTGTAGGACACTTTGT	-39.2
St3gal2	chro16, ex 2	GAGCGTGGCCATGCTGTGGTGCGAG	-30.4
Man1A	chro6, ex 5	AGAGTAGTCCACCAACAAAGCGTAT	-36.1
Standard control		CCTCTTACCTCAGTTACAATTTATA 10	

ASO: Antisense oligonucleotides; dG: the change in Gibbs free energy; chro: chromosome; ex: exon
