# Supplementary material for: Modulating aging-associated phenotypes in keratinocytes using antisense oligonucleotides targeting glycogenes
Source: Nanotheranostics. 2026 Oct 9;10:306–16. doi: 10.7150/ntno.135510 (PMC13686789; doi:10.7150/ntno.135510)
Supplement: Supplementary file 1 [file ntnov10p0306s1.pdf]

Supplementary data

Modulating Aging-Associated Phenotypes in Keratinocytes Using Antisense Oligonucleotides Targeting Glycogenes

*Bhanubhong Prommalee and Chalermchai Pilapong**

Laboratory of BioMolecular Imaging, Molecular and Cellular Biology, Department of Radiologic Technology, Faculty of Associated Medical Sciences, Chiang Mai University, Chiang Mai, Thailand

*E-mail: chalermchai.pilapong@cmu.ac.th. Phone: +66 53 94 9282. Fax: +66 53 21 3218; orcid.org/0000-0002-9099-3979

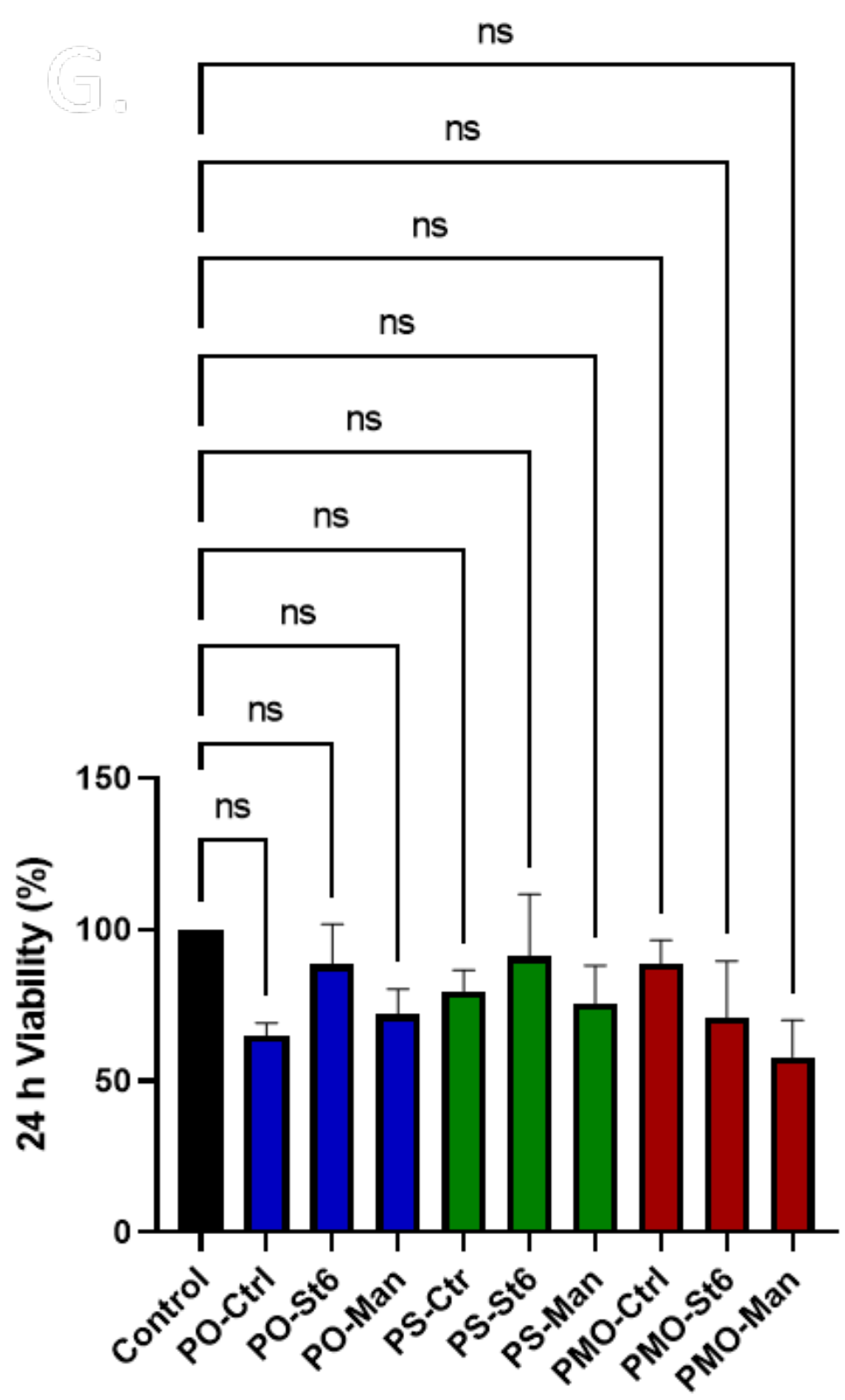

Figure S1. Comparison of cell viability following treatment with ASOs (1.0 nmol) containing different linkage chemistries at 24 h. Experiments were performed independently in triplicate (n = 3). ns, not significant (one-way ANOVA followed by Fisher's LSD post hoc test).

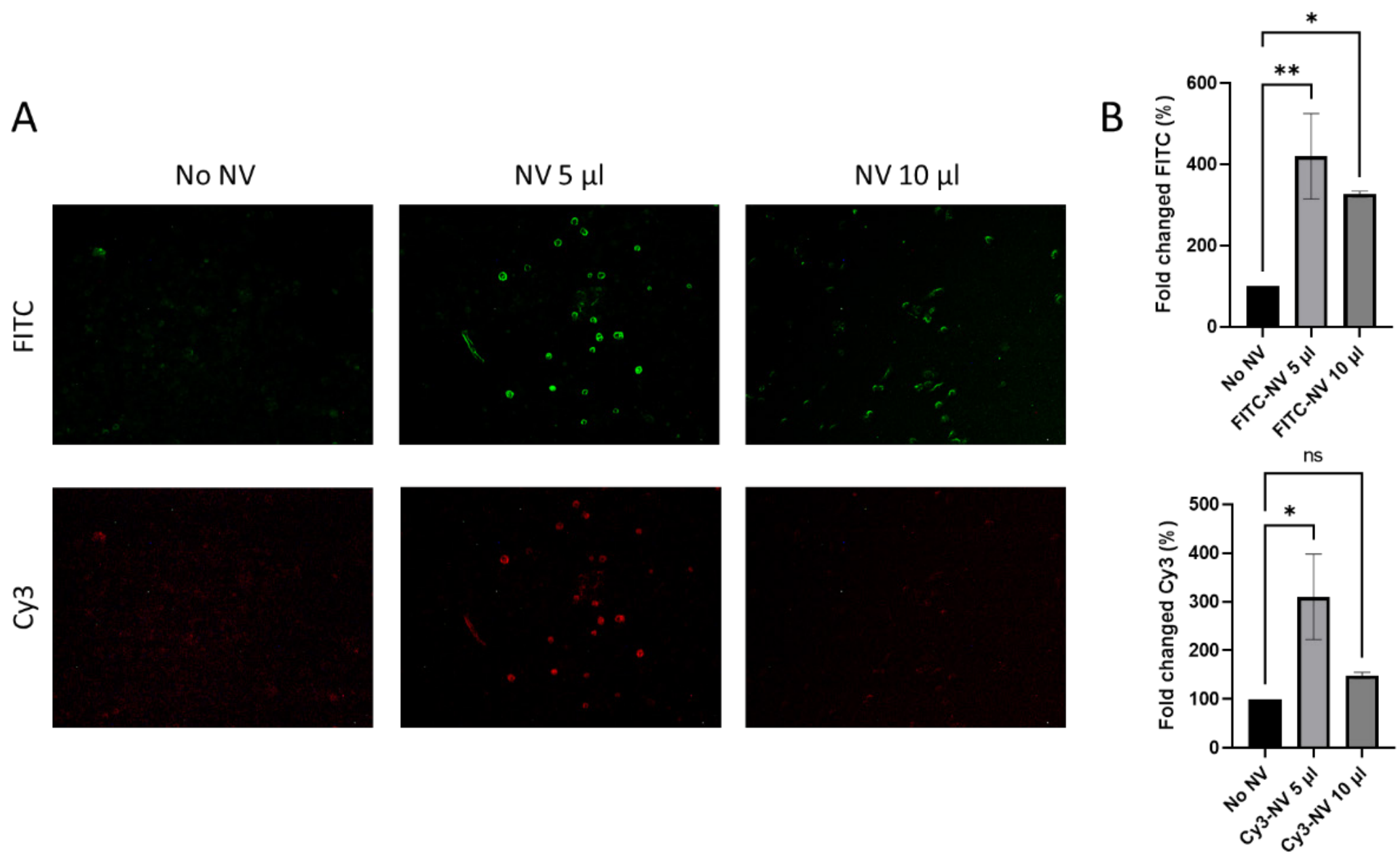

Figure S2. (A) Fluorescence imaging and (B) flow cytometric analysis of cells treated with Cy3-labeled ASOs (0.1 nmol) loaded onto different amounts of NV (No NV, 5 μL, and 10 μL). Cy3 fluorescence represents the ASO strand, whereas FITC fluorescence represents NV. Experiments were performed independently in triplicate ($n = 3$). ns, not significant, $*p < 0.05$ (one-way ANOVA followed by Fisher's LSD post hoc test).
